# Ultrasonic Modification of Ag Nanowires and Their Applications in Flexible Transparent Film Heaters and SERS Detectors

**DOI:** 10.3390/ma12060893

**Published:** 2019-03-18

**Authors:** Jie Sun, Xinxiang Yu, Zhutie Li, Junfeng Zhao, Pengcheng Zhu, Xiaoyan Dong, Zhigang Yu, Zhiguo Zhao, Dandan Shi, Junqin Wang, Han Dai

**Affiliations:** 1 Laboratory of Advanced Light Alloy Materials and Devices, Yantai Nanshan University, Longkou 265713, China; sunjie19860304@163.com (J.S.); yuxinxiangcn@163.com (X.Y.); zhaojunfengcc@163.com (J.Z.); lichkingoo@126.com (P.Z.); yuzhigang5101@163.com (Z.Y.); wjqing200@sohu.com (J.W.); 2 Postdoctoral Station of Nanshan Group Co., Ltd., Longkou 265706, China; 3 Hang Xin Material Technology Co. Ltd., Longkou 264006, China; lizhutie@nanshan.com.cn (Z.L.); zhaozhiguo@nanshan.com.cn (Z.Z.); shidandan@nanshan.com.cn (D.S.); 4 Nanshan Aeronautical College, Yantai Nanshan University, Longkou 265713, China; pigeon929@126.com

**Keywords:** Ag nanowires, ultrasonic modification, transparent film heaters, SERS

## Abstract

Ultrasonic morphology modification of silver (Ag) nanowires and their applications in transparent film heaters for defogging in electric vehicles and surface-enhanced Raman scattering (SERS) detectors have been studied. With 10 min ultrasonic treatment of Ag nanowires, the electro-thermal conversion capability of Ag nanowire based transparent film heaters is efficiently improved (about 50% increase in temperature rise), which can be mainly attributed to the cross-section area reduction and the serious agglomerations of the ultrasonic modified Ag nanowire films. Furthermore, the bending or fracture caused by deformation of Ag nanowires after ultrasonic treatment provides more hot spots for SERS, and therefore lead to a significant SERS signal enhancement. This work not only greatly improves the performance of Ag nanowire based transparent film heaters and SERS detectors, but provides a new way for the functional modification of Ag nanowires.

## 1. Introduction

Recently, Ag (silver) nanowires as flexible transparent conductive materials have been used for flexible or stretchable transparent heater for defogging, water heating, thin film actuation, bioheating, etc. [1,2,3,4]. Meanwhile, Ag nanowires are widely used in durable SERS detectors for their excellent optical and electrical properties [5]. At present, Ag nanowires can be prepared by various ways, such as deposition in hard templates, microwave, and hydrothermal synthesis [6,7,8]. All these methods provide long and straight Ag nanowires, due to the growth habits of metal nano-crystals, or limited by straight template, such as anodic aluminum oxide (AAO) et al. [9,10]. However, the build of nano-structure devices need bended or other Ag nanowires with complex topology, such as a shaped tip at the end of Ag nanowires could have higher thermal conversion efficiency and SERS signals. Due to the complex process and expensive equipment requirements in our previous study by a tip engineering technology on Ag nanowires [11], a simple way to induce topographic changes in Ag nanowires is urgently needed.

Ultrasonic treatment is often used to clean and purify Ag nanowires because of the PVP adhesion to Ag nanowires during the hydrothermal synthesis process [12,13]. Generally, ultrasonic cleaning for a short time with low power will not lead to obvious deformation of Ag nanowires due to the low probability of cavitation bubbles induced by the ultrasonic treatment [14]. However, long-duration or high-power ultrasonic treatments can both lead to surface corrosion for bulk material surfaces and cause serious deformation of nanostructures at the nanoscale [15,16]. This phenomenon provided us with the inspiration for the functional modification of Ag nanowires through ultrasonic treatments.

Herein, we employ a long duration low power ultrasonic treatment to modify the morphology of Ag nanowires and apply it in flexible transparent film heaters for defogging in electric vehicles and SERS detectors. The experiment results show that most Ag nanowires (average length 40 μm, diameter 100 nm) were bent and broken into fragments (length within 20 μm) with 10 min ultrasonic treatment (0.1 W/cm^2^). In contrast to the original morphology of the Ag nanowires, Ag nanowires after ultrasonic treatment exhibit excellent electro-thermal properties and enhance SERS signals.

## 2. Experiments

Ag nanowires (average diameter 100 nm, length 40 µm) were purchased from Nanjing XFNANO Materials Tech Co., Ltd. (Nanjing, China) Ag nanowires were dispersed in ethanol at 1 wt %. The ethanol suspension was sonicated in an ultrasonic cleaner for 10 min at 20 kHz. Then, dried at 80 °C for 1 h.

A mixture of poly (methyl methacrylate) (PMMA) was dissolved in tetrahydrofuran (THF). The THF solution was dripped and spin-coated onto glasses at 6000 rpm for 45 s. The film was dried at 80 °C for 1 h. Then, Ag nanowire (with and without ultrasonic treatment) ethanol suspension was dripped and spin-coated onto PMMA layer and was also dried at 80 °C for 1 h.

Specific reports suggest removing PMMA from Ag NWs/PMMA/glass could provide cleaner observation of the effects of the topography changes of Ag nanowires on the electro-thermal performances. The resistivity is characterized by the four-point probe sheet resistance method. Moreover, Cu electrodes were prepared on glass to measure the whole resistance of Ag nanowires (10 μL Ag nanowires, area 1 cm^2^) with and without ultrasonic treatment by a multimeter.

The morphology changes of Ag nanowires through ultrasonic treatment were characterized by optical microscope (ZEISS Image M2m, Dresden, Saxony, Germany), a transmission electron microscope (TEM, JEOL JEM 2100 LaB6, Tokyo, Japan) and a field emission scanning electron microscope (SEM, Hitachi UHR FE-SEM SU8010, Tokyo, Japan). The light absorption of the Ag nanowires dispersion solution was characterized by a UV spectrophotometer (Hitachi S4800, Tokyo, Japan). Finally, the SERS signals of rhodamine 6G (Rh6G, Sinopharm Chemical Reagent Co., Ltd., Beijing, China) on Ag nanowire films were detected by Raman spectrometer (LabRAM Aramis, Paris, France).

## 3. Results and Discussion

It is well known that ultrasonic treatment induced cavitation bubbles have enormous concentrations of energy, which is released intensely as bubble jets with flow rate up to 1000 m/s [17,18]. Such high flow rate can lead to pressures up to a GPa on nanowires [19]. When the Ag nanowires are exposed to ultrasonic bath, high intensity ultrasonic tends to break into small pieces [20]. Despite the low probability of cavitation bubbles induced by the ultrasonic treatment with low power in our case, the long duration treatment also provides abundant opportunity for the effects to act on these Ag nanowires [21]. As reported, the optoelectronic properties of Ag nanowires are based on their size and topography [22,23,24]. As a result, these properties of Ag nanowires should be changed after long duration ultrasonic treatments.

**Figure 1:**
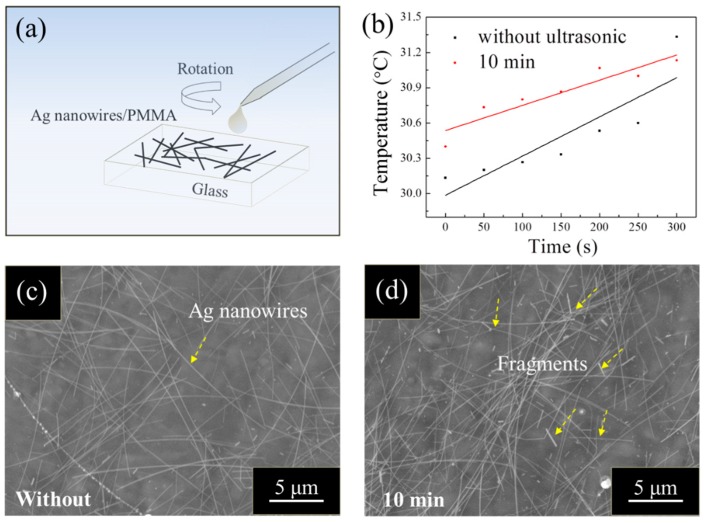
(**a**) A schematic of the structure of the Ag nanowires based film heater; (**b**) temperature rise of the Ag nanowires based film heaters; (**c**,**d**) top-views of Ag nanowires based film heater without and with ultrasonic treatment. Partial fragments of Ag nanowires were highlighted by yellow dot square frames.

**Figure 2:**
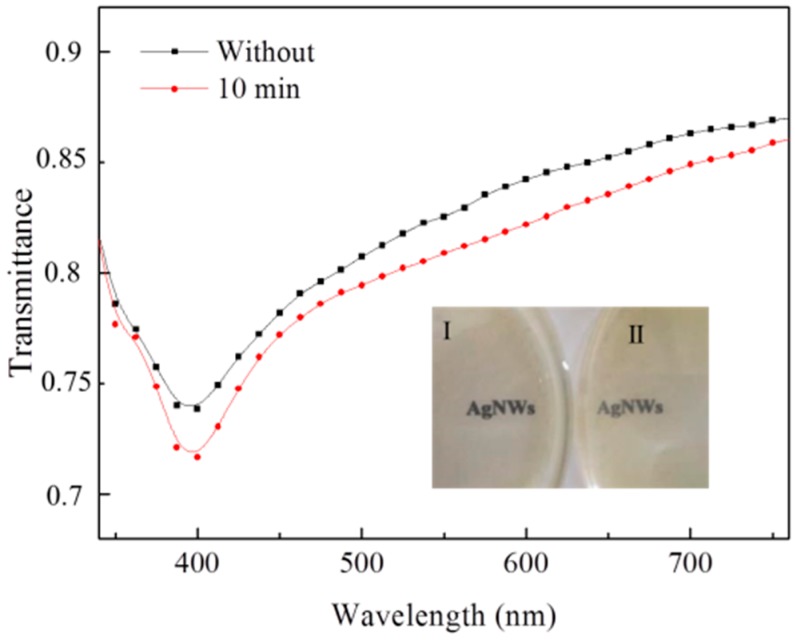
Transmittances of Ag nanowires before and after 10 min ultrasonic treatment. Inset image is the optic images of the Ag nanowire suspension before and after 10 min ultrasonic treatment.

Ag nanowire based PMMA film heaters were prepared (Ag NWs/PMMA/glass structure), as shown in Figure 1a. Ag nanowires with and without ultrasonic treatment were mixed with PMMA and were spin-coated on glass substrates. Glass substrate is very cheap, transparent, and its surface is very smooth. This makes it suitable materials for preparing PMMA/Ag nanowire layers. At the same time, it is also convenient for the subsequent temperature rise test. From the top view of these Ag nanowire based PMMA film heaters, Ag nanowires without ultrasonic treatment (Figure 1c) show a long and straight topography. In contrast to the original topography of Ag nanowires, Ag nanowires become bent and fractured after 10 min of ultrasonic treatment, as shown in Figure 1d. Moreover, significant agglomeration of Ag nanowires with ultrasonic treatment can also be observed in the heaters. The resistivity of Ag NWs/PMMA/glass in Figure 1c,d was characterized by the four-point probe sheet resistance method, as shown in Table 1. The average resistivity of the samples with ultrasonic treatment is 80.30 Ω/sq, which is clearly much higher than that without (35.39 Ω/sq). Thermocouples were fixed on the Ag NWs/PMMA/glass at three points, A (center), B (between the center and margin), and C (margin) to measure the temperature. LED sources (resistance about 90 Ω) have been incorporated into the circuit, which is used to avoid the short or open circuit in our work. When applying less than 6 volts, Ag nanowire-based heaters with 10 min ultrasonic treatment shows about a 50% increase in temperature rise compared to those without, as shown in Figure 1b. The raw data of the temperatures of Ag nanowire-based heaters with and without ultrasonic treatment are presented in Tables S1 and S2. However, slight transmittance reduction of these heaters in the visible spectrum was also observed after ultrasonic treatment, as shown in Figure 2. In order to better show the transmittance difference between them, Ag nanowire suspension (100 μL, 1 wt %) with and without ultrasonic treatment was presented in the inset image in Figure 2. As shown in this image, Ag nanowires without ultrasonic treatment show a clearer view of the characters ‘AgNWs’. Obviously, subjecting Ag nanowires to ultrasonic treatment can efficiently improve their electro-thermal conversion capability but it may lead to a slight transmittance reduction in transparent film heaters.

**Figure 3:**
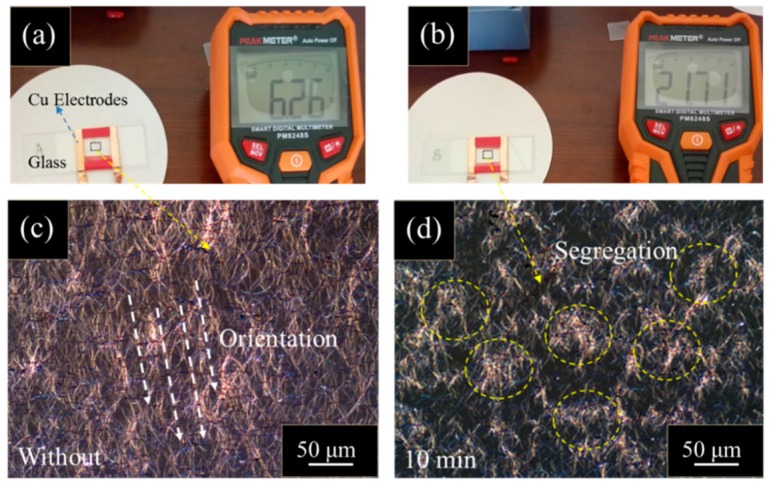
(**a**,**b**) Resistivity test of Ag nanowire films without and with 10 min ultrasonic treatment on glass substrates, respectively; (**c**,**d**) optic images of the Ag nanowire films without and with 10 min ultrasonic treatment, respectively. The agglomerations of Ag nanowires are marked by yellow dashed circles.

A verification resistivity test was carried out. As shown in Figure 3, Ag nanowires with and without ultrasonic treatment directly drops on the glass substrate (Ag NWs/glass structure). In contrast to Ag nanowires with their original topography, Ag nanowires with 10 min of ultrasonic treatment show about three times higher resistivity. The result can be ascribed to the differences in the size, topography, and arrangements between Ag nanowire films with and without ultrasonic treatment. As shown in Figure 3c and Figure S1, Ag nanowires without ultrasonic treatment shows long, uniform topography and ordered arrangement. However, Ag nanowires after ultrasonic treatment exhibit short, bending, and extensive agglomeration arrangements on glass substrate, presented in Figure 3d. Such agglomerations of Ag nanowires have also been observed in Ag nanowires based PMMA film heaters. Therefore, the electro-thermal conversion capability improvement can be mainly attributed to the changes in size, topography, and arrangement of Ag nanowires after ultrasonic treatment.

**Figure 4:**
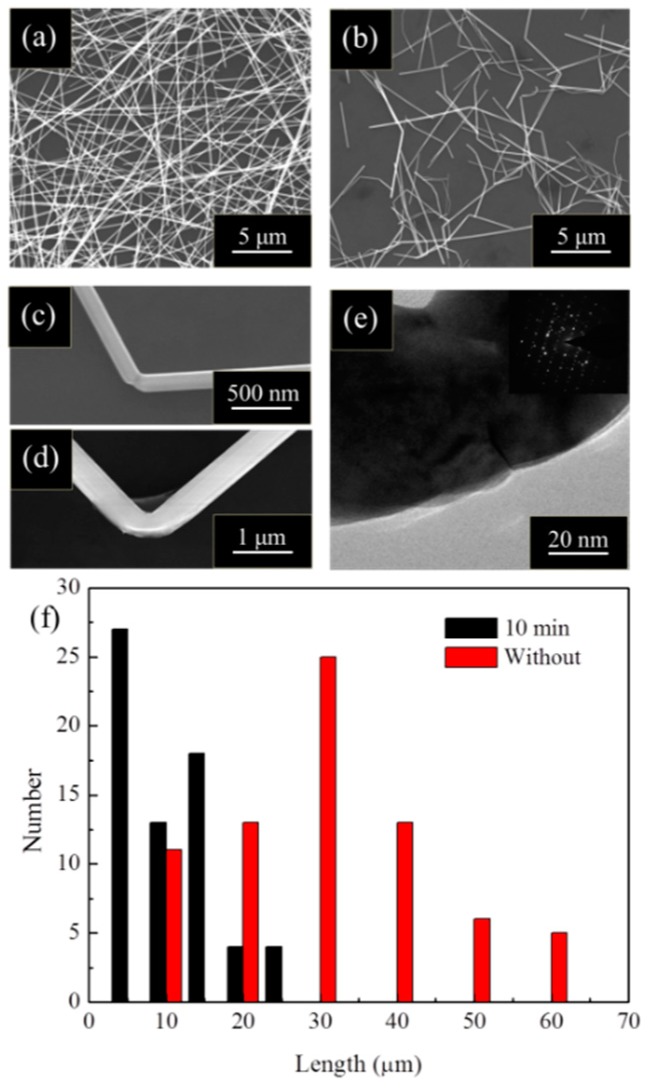
(**a**) Original morphology of Ag nanowires; (**b**) Ag nanowires after 10 min ultrasonic treatment; (**c**,**d**) bending deformation of Ag nanowire with different diameters; (**e**) TEM and selected area electron diffraction (SAED) on the bent area of Ag nanowire; (**f**) statistical results of the length of Ag nanowires before and after 10 min ultrasonic treatment.

To further explain ultrasonic treatment induced changes in size, topography, and arrangement of Ag nanowires on the electro-thermal conversion capability improvement of the heaters, Ag nanowires with and without ultrasonic treatment were characterized in detail. As shown in Figure 4a, Ag nanowires without ultrasonic treatment exhibit their characteristic long and straight topography. After a 10 min ultrasonic treatment, nearly all of the Ag nanowires were bent or fractured, as shown in Figure 4b. The statistical results of the lengths of the Ag nanowires were presented in Figure 4f, with the average length of the Ag nanowires reduced from about 42 nm to 16 nm after ultrasonic treatment. The resistance of Ag NWs/PMMA/glass can be divided into two parts: resistance of Ag NWs themselves and the contact resistances between Ag NWs. Apparently, the first part is much lower than that of the later. The contact resistance greatly increased as the average length of the Ag nanowires was reduced after ultrasonic treatment, because of the significant increment of the overlapping of Ag NWs. After ultrasonic treatment, the cross-section of the Ag nanowires (diameters of 150 nm and 400 nm) was greatly reduced (Figure 4c,d) because of the necking or surface breakage at the bending area respectively. As shown in Figure 4e, serious lattice distortions and a nano-crack can also be found at this location, which leads to serious electron scattering, thereby cause a significant rise in resistance. Owing to uneven multiple overlapping of the bent and fractured Ag nanowires, the contact resistance of the Ag nanowire conductive network grows higher than that of the Ag nanowires with their original topography. Therefore, the resistance of Ag nanowire with ultrasonic treatment should be much higher than that without. Moreover, this efficiently improves the electro-thermal conversion capability in transparent film heaters.

**Figure 5:**
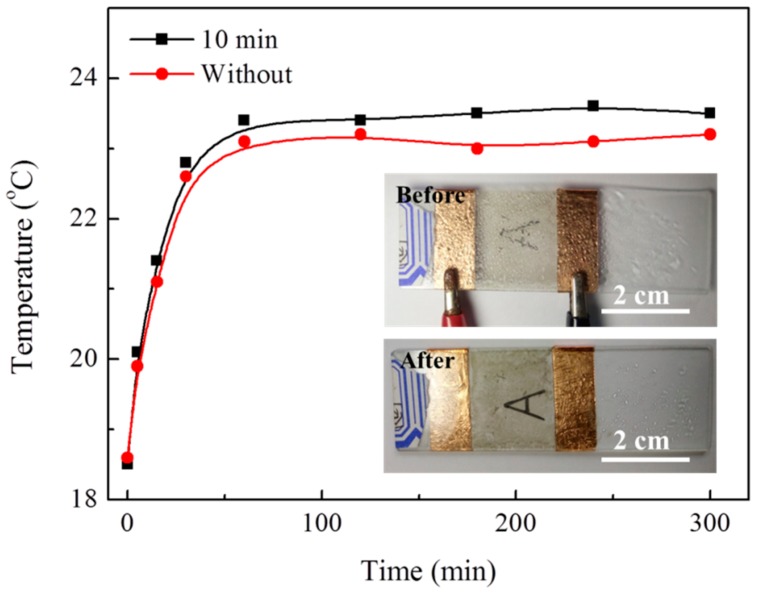
Temperature increase curves of Ag nanowire-based film heaters; inset images are the optical photograph of the defogging process on the Ag nanowires based film heater.

Ag nanowire-based film heaters can be applied on the windows of the electric vehicles for defogging. As reported, the driving distance will be reduced to half or even lower than before, when the tradition hot air is turned on for defogging [25]. This problem is particularly significant for electric vehicle under low temperature. As shown in Figure 5, the Ag nanowire-based film heaters increase the temperature to about 23 °C and successfully remove the fog on it by applying 10 V. Apparently, the thermal conversion efficiency of Ag nanowires with 10 min ultrasonic treatment is higher than that without. Due to the low Joule heat generated in Ag nanowires, this device exhibits stability even after continuous use for 100 h. As a result, the Ag nanowire-based film heaters have great potential application in electric vehicles for defogging.

**Figure 6:**
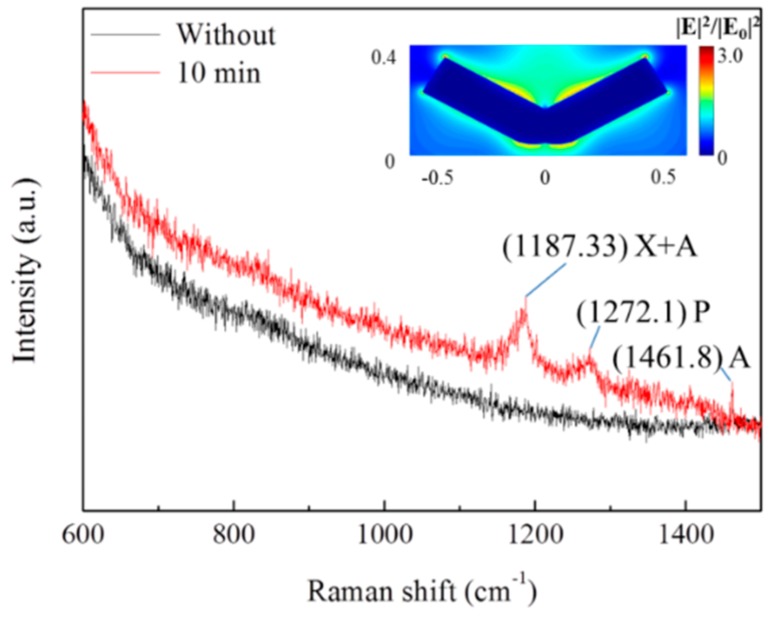
SERS signals of Ag nanowires before and after 10 min ultrasonic treatment.

Ultrasonic treatment leads to serious deformation of Ag nanowires and reduces their light transmittance. However, from view of the near field optics, Ag nanowires with bending deformation or fracture into fragments can efficient excite localized plasmon [26], which can provide many hot spots for SERS signals. The localized plasmon on Ag nanowire has been performed by FDTD simulations [11]. A model Ag nanowire 100 nm in diameter and 1 μm in length was adopted. A plane wave light source (wavelength of 538 nm) was used to illuminate the Ag nanowire. A top view of the cross-section of the Ag nanowire was presented in the inset image in Figure 6. Obvious light enhancement could be clearly observed at the bending area and the ends of Ag nanowires. As shown in Figure 6, a drop of Rh6G solution with a concentration of 10^−7^ M generated intense Raman signals on the Ag nanowire films. The signal from Rh6G at 1187, 1272, and 1462 cm^−1^ was remarkably increased by ultrasonic treatment. Moreover, similar results could be obtained in other parts of our samples, as shown in Figure S2. As a result, the ultrasonic morphologic modification of Ag nanowires provides an efficient way to enhance their SERS signals, which could be applied widely in Ag nanowire-based SERS detectors.

In summary, the ultrasonic morphologic modification of Ag nanowires and their applications in transparent film heaters on the windows of the electric vehicles for defogging and increases the SERS signals. Mainly owing to the cross-section area reduction and the serious agglomerations of the ultrasonic modified Ag nanowires, the electro-thermal conversion capability is efficiently improved in transparent film heaters. Moreover, ultrasonic treatment induced serious deformation of the Ag nanowires, such as bending deformation or fracturing into fragments, thus provides more hot spots for SERS and enhancing their SERS signals. Considering the wide application of Ag nanowire-based devices, this work provides an economical technique for the functional modification of Ag nanowires.

## Figures and Tables

**Table 1:** Resistivity of Ag NWs/PMMA/glass in Figure 1c,d without and with ultrasonic treatment, respectively.

	Resistivity (Ω/sq)									Avg.	
Without	30.63	41.58	34.51	33.98	31.85	39.91	32.24	39.98	36.95	32.25	35.39
With	61.32	57.78	136.23	68.44	63.07	60.53	78.78	73.66	148.76	54.38	80.30

## Supplementary Materials

The following are available online https://www.mdpi.com/1996-1944/12/6/893/s1. Figure S1: Optic image of the Ag nanowire films without ultrasonic treatment; Figure S2: SERS signals of Ag nanowires before and after 10 min ultrasonic treatment in other points; Table S1: Temperatures of A (center), B (between the center and margin), and C (margin) without treatment; Table S2: Temperatures of A (center), B (between the center and margin), and C (margin) with ultrasonic treatment.
