# Supplementary material for: Ultrasonic Modification of Ag Nanowires and Their Applications in Flexible Transparent Film Heaters and SERS Detectors
Source: Materials (Basel). 2019 Mar 18;12(6):893. doi: 10.3390/ma12060893 (PMC6471350; doi:10.3390/ma12060893)
Supplement: Supplementary file 1 [file materials-12-00893-s001.pdf]

materials

Supporting information

Ultrasonic modification of Ag nanowires and their applications in flexible transparent film heaters and SERS detectors

Jie Sun [1], Xinxiang Yu [1,2], Zhutie Li [3], Junfeng Zhao [1], Pengcheng Zhu [1], Xiaoyan Dong [4], Zhigang Yu [1], Zhiguo Zhao [3], Dandan Shi [3], Junqin Wang[1] and Han Dai [1,2,*]

[1] Laboratory of Advanced Light Alloy Materials and Devices, Yantai Nanshan University, Longkou 265713, China;
[2] Postdoctoral Station of Nanshan Group Co., Ltd.;
[3] Hang Xin Material Technology Co. Ltd., Longkou 264006, China;
[4] Nanshan Aeronautical College, Yantai Nanshan University, Longkou 265713, China;
* Correspondence: daihan1985@189.cn; Tel.: +86-0535-859-0938 or +86-0535-860-9860

Figures

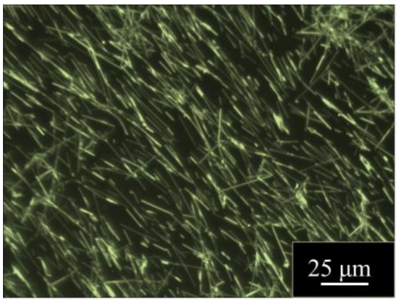

Figure S1. Optic images of the Ag nanowire films without ultrasonic treatment.

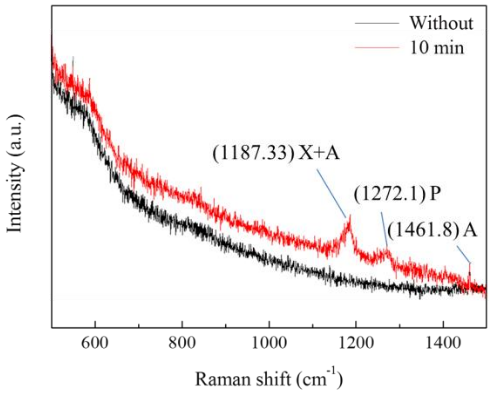

Figure S2. SERS signals of Ag nanowires before and after 10 min ultrasonic treatment in other point.

Tables

Table S1. Temperatures of A (Centre), B (Between centre and margin), C (Margin) without treatment.

Time(s)	A(°C)	B(°C)	C(°C)	Avg.(°C)
0	30.40	30.40	30.40	30.40
50	30.60	30.80	30.80	30.73
100	30.80	30.80	30.80	30.80

150	31.00	30.80	30.80	30.87
200	31.20	31.20	30.80	31.07
250	31.00	31.00	31.00	31.00
300	31.20	31.20	31.00	31.13

Table S2. Temperatures of A (Centre), B (Between centre and margin), C (Margin) with ultrasonic treatment.

**Time(s)**	**A(°C)**	**B(°C)**	**C(°C)**	**Avg.(°C)**
0	30.00	30.20	30.20	30.13
50	30.20	30.00	30.40	30.20
100	30.40	30.00	30.40	30.27
150	30.40	30.20	30.40	30.33
200	30.60	30.40	30.60	30.53
250	30.60	30.40	30.80	30.60
300	31.40	31.20	31.40	31.33
